# Cancer related cognitive impairment: a downside of cancer treatment

**DOI:** 10.3389/fonc.2024.1387251

**Published:** 2024-04-23

**Authors:** Kimberly Demos-Davies, Jessica Lawrence, Davis Seelig

**Affiliations:** ^1^ Department of Veterinary Clinical Sciences, University of Minnesota College of Veterinary Medicine, Saint Paul, MN, United States; ^2^ Masonic Cancer Center, University of Minnesota, Minneapolis, MN, United States

**Keywords:** cancer related cognitive impairment, CRCI, cancer treatment, chemobrain, neurobehavior

## Abstract

Cancer treatment is associated with long lasting cognitive impairment in cancer survivors. This cognitive impairment is often termed cancer related cognitive impairment (CRCI). Cancer survivors treated for tumors outside the central nervous system are increasingly diagnosed with CRCI. The development of strategies to mitigate the negative effects of cancer treatment on the brain are crucial. Although neuroimaging research has proposed several candidate mechanisms, the pathogenic underpinnings of CRCI remain uncertain. As such, preventative and treatment strategies have not been identified. To fill these gaps, animal models play a vital role in isolating underlying contributing mechanisms that promote CRCI and in testing new therapeutic approaches.

## 1 Introduction

There are an estimated 18.1 million cancer survivors in the United States as of 2022 (1). Advances in early screening techniques and cancer treatment have extended cancer patients survival (2–5). With this increased longevity of cancer patients, the importance of quality of life is a vital survivorship issue (2, 4). Among the adverse effects of cancer treatment is a syndrome of cognitive impairment known as cancer related cognitive impairment (CRCI) (5). Cancer survivors affected by CRCI report visual and verbal memory impairment, learning and attention impairment, and difficulty processing new information and multitasking (6–8). CRCI decreases cancer survivors’ quality of life by negatively impacting their daily tasks and personal relationships, negatively impacting work performance and, decreasing their ability to return to work (9–12). This review will cover the proposed mechanisms for CRCI in cancer patients and the animal models being used to determine potential therapeutic targets.

## 2 Cancer related cognitive impairment

Cancer related cognitive impairment affects cancer patients with non-central nervous system cancers before, during and after treatment (2, 13). The combined adverse effects of both cancer and cancer treatment cause CRCI (11, 13). The cancer treatments implicated in cognitive impairment include chemotherapy, radiation therapy, surgery, endocrine therapy, and immunotherapy (8, 11, 13). CRCI is estimated to effect 75% of cancer patients for durations up to 10 years following treatment for solid tumors including breast, lung, intestinal, ovarian, prostatic and testicular tumors (5, 7). CRCI is documented most thoroughly to date in women with breast cancer (BC), which represents 22% of all cancer survivors in the United States (2). BC survivors have reported cognitive dysfunction for 5-10 years following treatment with chemotherapy and/or radiation therapy (7, 14).

The diagnosis of CRCI is challenging as there is no clear consensus on a diagnostic algorithm best applying the numerous measures that assess cognitive function (13). The International Cancer and Cognitive Task Force (ICCTF) established criteria for determining cognitive impairment in cancer patients and recommends the use of neuropsychological tests that are sensitive for the cognitive domains most impaired by cancer treatment (15). Such neuropsychological objective tests remain the gold standard to objectively assess cognitive impairment (15, 16). Specifically, the ICCTF recommends the Hopkins Verbal Learning Test-Revised (HVLT-R), the Trail Making Test (TMT) and the Controlled Oral Word Association (COWA) or the Multilingual Aphasia Examination (16). These tests measure learning, memory, attention, processing speed and executive function, which are the principal cognitive domains affected in CRCI (16). These neuropsychological tests in cancer patients fail to reliably correlate with patients’ clinical cognitive impairment (15). This gap between neuropsychological test performance and self-reported clinical signs may result from compensatory activation of unaffected brain regions in cancer survivors to preserve performance on neuropsychological testing (15). Alternatively, patient self-reported measures, including the Functional Assessment of Cancer Therapy-Cognitive (FACT-Cog) have been shown to more strongly associate with the symptoms reported by cancer patients compared to their performance on objective neuropsychological testing (2, 15).

Standard neuroimaging in patients suffering from CRCI is frequently unremarkable (2, 17). Advanced imaging studies using volumetric magnetic resonance imaging (MRI), diffusion tensor imaging (DTI) and positron emission tomography have shown widespread reductions in gray matter volume, changes in white matter connectivity, alterations in brain activation and neuroinflammation (activation of microglia and astrocytes) in cancer patients with CRCI (2, 4, 5, 18, 19).

### 2.1 Chemotherapy

Chemotherapy has been implicated in causing CRCI, with terms such as “chemobrain” or “chemofog” often used to refer to this syndrome (7, 20–23). The cognitive domains affected in chemotherapy-associated CRCI include learning, memory, attention, executive function, language and processing speed (2, 8). In longitudinal studies, the incidence of CRCI after chemotherapy is reported from 17% to over 70% among cancer patients (2). Known risk factors that increase incidence of CRCI after chemotherapy include non-cancer related comorbidities, anemia, and genetic predisposition (24, 25). The proposed mechanisms for chemotherapy-associated CRCI include decreased neurogenesis, axonal degeneration and demyelination, reduction in dendrite volume and length, increased oxidative stress and free radical formation, neuroinflammation, toxicity to CNS progenitor cells, microglia apoptosis, blood brain barrier disruption, brain blood flow alterations, myelin degradation, production of damage associated molecular patterns, cytokine-induced neurotoxicity, reduced glucose metabolism, alteration in brain water content, and fluctuations in neurotransmitter levels (5, 14, 22, 23, 25, 26). Select chemotherapy drugs, including 5-fluorouracil (5-FU), carmustine (BCNU), lomustine (CCNU), temozolomide (TMZ) cross the intact blood brain barrier to directly injure the brain. Additional drugs such as methotrexate (MTX), paclitaxel, docetaxel (DTX), cisplatin may cross in small concentrations to negatively affect brain tissue (11, 22, 25–27). However, for drugs like doxorubicin (DOX), known to be associated with CRCI, it is unclear how it alters neural cells. Chemotherapy-associated CRCI is often mild to moderate in severity and can be dependent on the chemotherapy agent received and the dose (2, 8, 28). Chemotherapy agents such as MTX, BCNU, fludarabine, cytarabine and 5-FU can cause severe neuropathological diseases including encephalopathy and dementia (2).

The bulk of our understanding of chemotherapy-associated CRCI stems from studies of cognitive impairment in BC survivors during and after chemotherapy treatment (summarized in **Table 1**) (19, 29, 64, 80–82). BC patients after chemotherapy treatment report deficits in cognitive domains consisting of executive function, working memory, attention, concentration, processing speed, cognitive flexibility, visual and verbal memory (83). Various structural brain changes have been described in these BC patients, including reduction in gray matter, decreases in white matter integrity, altered brain activation, neuroinflammation and changes in brain functional connectivity (4, 8, 15, 18, 28, 84–86). PET imaging acquired 5-10 years after treatment in BC patients demonstrated altered frontal cortex activation during a short term verbal memory task in survivors treated with chemotherapy compared to survivors not treated with chemotherapy (84). One study used PET-MR imaging and neuropsychological testing to demonstrate that compared to patients treated with surgery alone, BC patients treated with surgery and adjuvant chemotherapy had higher brain local translocator protein expression, a measure of neuroinflammation that was associated with worse cognitive performance (4).

**Table 1:** Summary of findings from clinical studies evaluating cancer treatments effect on cognitive function.

Short term follow up studies				
Reference	Type of cancer	Treatment	Time post treatment	Cognitive function
Jim et al., 2009 (20)	Stage I, II and ductal carcinoma *in situ* BC	Multiagent CT (including two or more of the following: DOX, CYP, taxotere, paclitaxel, MTX, 5-FU and epirubicin) ± hormonal therapy	6 months	Impairment in episodic memory and attention compared to healthy age matched control women
		ECRT± hormonal therapy	6 months	Impairment in attention and complex cognition compared to healthy age matched control women
Shilling et al., 2005 (21)	Early BC	Multiagent CT (5-FU + epirubicin + CYP or CMF or FU + epirubicin + CYP + DTX)	18 months	Impairment in concentration and attention after CT compared to before CT 78% reported memory problems during CT 2.25 times as likely as the control healthy women to be classified as having cognitive impairment
Williams et al., 2018 (29)	Stage I-IV BC	Non-anthracycline-based CT or anthracycline-based CT	After cycle 2 of CT	Association between increasing serum soluble TNF receptor I and receptor II concentration and declines in short-term visual memory
Rodríguez et al., 2020 (30)	Stage I-IIIA BC	Received CT	2 months	Impairment in processing speed and attention compared to before CT was started
Cheung et al., 2015 (31)	Stage I-III BC	CT (DOX + CYP or CYP + DTX)	3 weeks	Higher concentrations of IL-1β and IL-6 were associated with more severe self-perceived cognitive impairment and decreased response speed performance
Schagen et al., 1999 (32)	BC	CT (CMF) ± hormonal therapy (tamoxifen)	2 years	Reported impairment in concentration and memory significantly more often than age matched patients that did not receive CT Impairment in attention, concentration, processing speed, mental flexibility, motor function and visual memory compared to age matched patients that did not receive CT
van Dam et al., 1998 (33)	Stage II and III BC	Multiagent CT (5-FU + DOX + CYP followed by CYP + thiotepa + carboplatin) + hormonal therapy (tamoxifen) + ECRT	2 years	Reported more cognitive problems compared to patients who did not receive CT Impairment in attention, concentration, processing speed, visual memory, and motor function compared to patients who did not receive CT
Wieneke et al., 1995 (34)	Stage I and II BC	Multiagent CT (CMF and/or CYP + DOX + 5-FU) ± hormonal therapy (tamoxifen)	At least 2 weeks	Impairment in attention, concentration, verbal and visual memory, mental flexibility, processing speed memory, visuospatial ability and motor function compared to aged matched, education status and gender matched controls
Fan et al., 2005 (35)	BC	Multiagent CT (CYP + epirubicin + 5-FU or DOX + CYP) ± tamoxifen ± ECRT	1 year	More BC patients treated with chemotherapy had moderate-severe cognitive dysfunction compared to healthy age matched controls
Stewart et al., 2008 (36)	Stage I-III BC	Multiagent CT (CYP + epirubicin + 5-FU or 5-FU + DOX + CYP or DOX + CYP or DOX + CYP + taxol or epirubicin + CYP + taxol or DOX + cisplatin) ± tamoxifen	30 days	Impairment in working memory compared to patients treated with only hormonal therapy
Fosså et al., 2003 (37)	Metastatic TC	CT (BEP)	2 years	20% reported worse cognitive function
Wefel et al., 2014 (38)	TC (non-seminomatous germ cell tumors)	Multiagent CT(two or more of the following: bleomycin, etoposide, cisplatin, DOX, paclitaxel, CYP, vinblastine, MTX, vincristine, ifosfamide)	1 year	Decline in psychomotor speed, learning and memory was more common in patients who received more cycles of CT Greater overall cognitive decline compared to patients that did not receive CT
Amidi et al., 2017 (39)	Stage I-III TC	CT (BEP)	6 months	Increased overall cognitive decline compared to patients that did not receive CT Changes in global and local network measures in the brain compared to before CT treatment and the patients that did not receive CT
Skaali et al., 2011 (40)	TC	CT (Carboplatin or BEP)	1 year	Increased self-reporting of cognitive impairment compared to patients that did not receive CT
Trachtenberg et al., 2018 (41)	Hodgkin’s lymphoma	CT (DOX + bleomycin + vinblastine + dacarbazine ± ECRT or bleomycin + etoposide + DOX + CYP + vincristine + procarbazine) ± ECRT	6 months - 5 years	Reported more frequently and more severe cognitive impairment than healthy controls 30% of patients were impaired in ≥ 2 cognitive domains including memory and executive function
Fayette et al., 2023 (42)	Hodgkin’s lymphoma	CT (DOX + bleomycin + vinblastine + dacarbazine and/or bleomycin + etoposide, DOX, CYP + vincristine + procarbazine) + ECRT	6 months	Decreased cognitive performance in the domains of verbal memory, learning and executive function compared to healthy controls
Zimmer et al. (43)	B-cell non- Hodgkin’s lymphoma	CT (R-CHOP)	3 months	Reported cognitive impairment
		Rituximab + CT(bendamustine)	3 months	Significantly lower scores on objective cognition test than healthy controls and patients treated with R-CHOP
Janelsins et al., 2022 (44)	Hodgkin’s and non-Hodgkin’s lymphoma	CT (R-CHOP or Rituximab + bendamustine or DOX + bleomycin + vinblastine + dacarbazine)	6 months	Significantly lower scores on neuropsychological testing in the domains of executive function and memory
Noal et al., 2011 (45)	BC	ECRT	1 year	Mild cognitive impairment
		CT (5-FU + epirubicine + CYP ± DTX)+ ECRT	1 year	Mild cognitive impairment
Shibayama et al., 2014 (46)	BC	ECRT ± CT ± hormonal therapy	7 months	Lower scores on memory testing compared to BC patients that did not receive ECRT
Donovan et al., 2005 (47)	Early stage BC	ECRT + CT (two or more of the following: DOX, CYP, Taxotere, paclitaxel, MTX and 5-FU)	6 months	No significant difference in cognitive impairment compared to BC patients that received ECRT alone
		ECRT	6 months	No significant difference in cognitive impairment compared to patients that received CT and ECRT
Kohli et al., 2007 (48)	breast, lung cancer, prostate cancer, hematological, gastrointestinal or head and neck cancer	CT	6 months	Self-reported problems with memory and concentration
		CT + ECRT	6 months	Self-reported problems with memory and concentration
		ECRT	6 months	Self-reported problems with memory and concentration
Thiery-Vuillemin et al., 2018 (49)	Prostate cancer (Adenocarcinoma)	Endocrine therapy (Enzalutamide)	3 months	Reported worse cognitive impairment compared to patients treated with abiraterone acetate plus prednisone
Bender et al., 2006 (50)	Stage I, II BC	CT	1 year	Impairment in verbal working memory
		CT + hormonal therapy (tamoxifen)	1 year	Impairment in visual memory and verbal working memory Reported more memory complaints than patients that did not receive tamoxifen
Scheibel et al., 2004 (51)	Chronic myelogenous leukemia	CT + immunotherapy (INF-α)	During treatment	Impairment in processing information and executive functions
Rogiers et al., 2020 (52)	First-generation metastatic melanoma	Immunotherapy (pembrolizumab)	≥6 months	Reported cognitive impairment 32% revealed cognitive impairment on neurocognitive testing
Rogiers et al., 2020 (53)	Metastatic melanoma	Immunotherapy (ipilimumab)	≥2 years	41% of the patients had cognitive impairment
Belin et al., 2020 (54)	B-cell lymphoma	Immunotherapy (C19-targeted CAR T-cell)	14 days	43% of patients had at least one neurological symptom after treatment Cognitive signs were severe in 36% of the patients
Ruark et al., 2020 (55)	Relapse/refractor chronic lymphocytic leukemia, non-Hodkin lymphoma, acute lymphoblastic leukemia	Immunotherapy (CD19-targeted CAR-T cells)	1-5 years	38% reported cognitive difficulties after treatment
Hoogland et al., 2022 (56)	Non-Hodgkin’s lymphoma	Immunotherapy (CAR-T-cell therapy)	1 year	35% of patients had scored at least 2 standard deviations below the mean on at least 1 neurocognitive test Delayed memory was the domain most impaired in the patients Visuospatial ability declined overtime in the patients post treatment
Myers et al., 2023 (57)	Head and neck squamous cell carcinoma, hepatocellular, melanoma, non-small cell lung cancer, renal cell, squamous cell carcinoma of orbit or urothelial cancer	Immunotherapy (First-line check-point inhibitors)	6 months	Neurological performance was significantly decreased compared to age-matched healthy controls. Reported cognitive impairment
Friedman et al., 2009 (58)	Hematopoietic malignancies	Immunotherapy (HCT)	6 weeks	Impairment in verbal learning
Nakamura et al., 2021 (59)	Acute leukemia	Immunotherapy (Allogenic HCT)	6 months	36% of patients met the criteria for at least mild cognitive impairment by neurocognitive testing
Schulz-Kindermann et al., 2007 (60)	Hematopoietic malignancies	Immunotherapy (Allogenic HCT)	3 months	Impairment in reaction time
Jones et al., 2013 (61)	Multiple myeloma	Immunotherapy (Allogenic HCT)	3 months	48% of patients showed cognitive decline on ≥ 1 measure on neuropsychological tests Impairment in learning, memory and psychomotor speed
Scherwath et al., 2013 (62)	Hematopoietic malignancies	Immunotherapy (Allogenic HCT)	1 year	41% of patients showed impairment in ≥ 1 cognitive domain Decline was seen in word fluency, verbal delayed recall, and verbal recognition compared to before treatment
Harder et al., 2007 (63)	Hematopoietic malignancies	Immunotherapy (HCT)	At least 1 year	Mild decline in attention, executive function and psychomotor function compared to patients who did not receive HCT

Long term follow up studies				
Reference	Type of cancer	Treatment	Time post treatment	Cognitive function
Kesler et al., 2013 (64)	Stage I-IIIA BC	Multiagent CT (DOX + CYP + paclitaxel or CYP + 5-FU + paclitaxel + MTX)	3.4 years	Impairment in verbal memory compared to age-matched healthy women Decreased verbal memory performance was associated with increased serum TNFα and decreased left hippocampal volume
Koppelmans et al., 2012 (65)	BC	Multiagent CT (CMF)	20 years	Impairment in verbal memory, processing speed, executive function and psychomotor speed compared to age matched healthy women
Yamada et al., 2010 (66)	Stage I-IIIA BC	Multiagent CT (CMF or DOX)	At least 10 years	Impairment in attention, working memory, psychomotor speed and executive function compared to age and educational matched healthy controls
van der Willik et al., 2018 (67)	BC	Multiagent CT (CMF) + ECRT	20 years	Lower global cognitive performance and higher inflammatory markers compared to healthy women Association between higher inflammatory markers and lower cognitive performance
Ahles et al., 2002 (68)	Stage II-IV BC or lymphoma	CT (one or more of the following: CYP, MTX, 5-FU, DOX, vincristine, carboplatin, mechlorethamine, procarbazine, bleomycin, dacarbazine, vinblastine, etoposide)	5 years	Impairment in verbal memory and psychomotor compared to patients that did not receive CT Reported greater changes in memory function compared to patients that did not receive CT
Schagen et al., 2008 (69)	TC	CT (BEP)	3 years	Over 50% of the patients reported cognitive complaints and 77% thought cognitive complaints was related to treatment. Percentage of patients classified as cognitive impairment was significantly greater than patients that did not receive CT
		ECRT	3 years	Percentage of patients classified as cognitive impairment was significantly greater than patients that did not receive ECRT
Pedersen et al., 2009 (70)	TC	CT (BEP)± ECRT	2-7 years	Patients did not show significant worse cognitive impairment compared to patients treated with ECRT and surgery
Chovanec et al., 2018 (71)	TC (germ cell tumors)	Multiagent CT (two or more of the following: bleomycin, etoposide, cisplatin, CYP, DOX, paclitaxel, OXP, ifosfamide, vinblastine, vincristine, dactinomycin)	10 years	Impairment in cognition compared to patients treated with surgery alone
		ECRT	10 years	Impairment in cognition compared to patients treated with surgery alone
		Multiagent CT + ECRT	10 years	Impairment in cognition compared to patients treated with surgery alone
Skoogh et al., 2012 (72)	Non-seminomatous TC	Multiagent CT (cisplatin + two or more of the following: vinblastine, bleomycin, etoposide, ifosfamide, mesna)	3-26 years since diagnosis	Increased incidence of long-term language impairment in survivors who received five or more cycles of cisplatin-based CT compared to survivors that did not receive CT
Stouten-Kemperman et al., 2015 (73)	TC	CT (BEP)	14 years	Lower cognitive performance compared to patients treated with surgery only Reported more memory problems after CT Significant changes in white matter microstructure compared to patients treated with surgery only
Amidi et al., 2015 (74)	Grade I-IV TC	± CT (BEP) ± ECRT	2-7 years	Cognitive impairment was found in the following domains: verbal learning and memory, visual learning and memory, processing speed, executive functioning, attention and working memory in patients treated with CT
Stelwagen et al., 2021 (75)	Stage I-IV TC	Platinum-based CT	>20 years	Performed worse than age matched healthy controls on cognitive function tests
		ECRT	>20 years	Performed worse than age matched healthy controls on cognitive function tests
Phillips et al., 2012 (76)	Stage 0-II BC	ECRT + CT (2 or more of the following: DOX, CYP, Taxotere, paclitaxel, MTX, 5-FU, epirubicin) ± hormonal therapy	3 years	Impairment in executive function and processing speed compared to healthy age matched controls No significant cognitive function differences compared to patients who received ECRT alone
		ECRT ± hormonal therapy	3 years	Impairment in executive function and processing speed compared to healthy age matched controls No significant cognitive function differences compared to patients who received ECRT and CT
Castellon et al., 2009 (77)	Stage 0-II BC	CT (CMF, DOX + CYP + taxane, or DOX + CYP)	2-5 years after diagnosis	Performed significantly worse on neuropsychological testing that assessed domains of verbal learning, visuospatial functioning and visual memory compared to patients who did not receive systemic therapy
		CT + hormonal therapy (tamoxifen)	2-5 years after diagnosis	Scored lower on the global neurocognitive performance measure compared to patients who did not receive systemic therapy
Sharafeldin et al., 2018 (78)	Hematopoietic malignancies	Allogenic HCT or autologous HCT	3 years	Cognitive impairment was present in 18.7% of autologous HCT patients and 35.7% of allogenic HCT patients Impairment of visual memory in the autologous HCT patients Impairment in executive function, verbal fluency and working memory in the allogenic HCT patients
Syrjala et al., 2011 (79)	Hematopoietic malignancies	Allogenic HCT	5 years	38% of patients had mild cognitive impairment Impairment in verbal learning and retention

Short term follow up studies included studies that evaluated cognitive impairment ≤ 2 years post treatment. Long term follow up studies included studies that evaluated cognitive impairment > 2 years post treatment. BC, breast cancer; CT, chemotherapy; ECRT, extracranial radiation therapy; TC, testicular cancer; DOX, doxorubicin; CYP, cyclophosphamide; MTX, methotrexate; 5-FU, 5-floururacil; DTX, docetaxel; CMF, cyclophosphamide, methotrexate, 5-floururacil; BEP, bleomycin, etoposide, cisplatin; RCHOP, Rituximab + CYP + DOX + vincristine + prednisone; OXP, oxaliplatin; HCT, hematopoietic stem cell transplantation.

Mechanistically, numerous studies have implicated systemic inflammation as a major contributor to chemotherapy-associated CRCI in BC patients. Chemotherapy associated inflammation is a potential significant contributor to CRCI; a thorough review of this subject is beyond the scope of the paper but has been well described in recent years (15, 80, 81). Chemotherapy agents have been reported to elevate multiple pro-inflammatory markers including interlukin-6 (IL-6), IL-1 beta (IL-1β), IL-5, IL-10, reactive oxygen species (ROS), IL-8, interferon gamma (IFN-α), IL-12, IL-17, and tumor necrosis factor alpha (TNF-α) (15, 82). Cognitive performance in chemotherapy-treated BC patients have specifically been linked to serum inflammatory markers (11, 29, 30, 64). One study found an association between increased concentrations of soluble tumor necrosis factor receptor I and II and decline in visual memory (29). Another study demonstrated a significant interaction between serum IL-6, TNF-α levels and verbal memory difficulties in BC patients treated with chemotherapy (64). In BC patients, one study showed that higher elevations in IL-6 and IL-1β were associated with more severe cognitive impairment (31) These studies support that select chemotherapy agents increase circulating inflammatory markers which subsequently cross the blood brain barrier to activate microglia and induce neuronal cell death (23, 87).

Several CRCI studies have evaluated the long-term impact of the adjuvant chemotherapy protocol CMF that includes cyclophosphamide (CYP), MTX and 5-FU for early-stage breast carcinoma (32). One study evaluated cognitive impairment via neuropsychological testing and self-reporting in BC patients 21 years following adjuvant CMF chemotherapy (65). These survivors performed worse on cognitive tests of learning, verbal memory, information processing speed, inhibition and psychomotor speed compared to non-cancer-bearing women (65). BC survivors also reported memory complaints that were not associated with neuropsychological testing, a commonly reported phenomena in other studies (20, 32, 33, 65). One study compared cognitive impairment at 2 years in BC survivors treated with surgery and adjuvant CMF and age-matched BC survivors treated with surgery only (32). In this study, CMF-treated survivors reported significantly more difficulties with memory and concentration and increased incidence of cognitive impairment than BC patients treated with surgery alone (32). An additional study evaluated stage I and II breast carcinoma patients 3-18 months after CMF treatment and found that 75% of patients scored 2 standard deviations below test norms (corrected for age, education and gender) on one or more neuropsychological tests (34). Another study showed that BC survivors treated with CMF at least 10 years prior had significant impairment in cognitive domains of executive function, working memory and divided attention compared to age, education status matched non-cancer controls (66). The underlying mechanisms leading to altered cognition are not clear. However, one study showed that 20 years after adjuvant CMF treatment, BC survivors had increased levels of systemic inflammation (increased granulocyte count to lymphocyte count ratio (GLR), increased platelet count to lymphocyte count ratio and increased platelet count times GLR) and lower cognitive performance compared to healthy controls (67).

While CMF-treated BC patients have provided abundant information regarding this syndrome, CRCI has also been documented and evaluated following administration of many adjuvant chemotherapy protocols. Chemotherapy-treated BC patients (most of whom received 5-FU, DOX and CYP) in one study were more likely to show cognitive impairment on neurophysiological tests than healthy women controls (21). In another study, BC survivors treated with a wide variety of multi-agent treatment protocols incorporating DOX, CYP, DTX, paclitaxel, MTX, 5-FU or epirubicin, demonstrated deficits in episodic memory as compared to age- and sex-matched non-cancer controls 6 months following treatment (20). One-to-two years following treatment with either DOX and CYP or CYP, epirubicin and 5-FU, significantly more BC survivors demonstrated moderate to severe cognitive impairment compared to age, sex, and educational status matched non-cancer controls (35). A recent prospective study showed that BC patients treated with a diverse repertoire of multi-agent chemotherapy protocols were at a threefold greater risk of cognitive decline compared to BC patients treated with hormone therapy (36). Notably, a prospective study showed that cognitive impairment in chemotherapy-treated BC patients was worse in the presence of anxious or depressive symptoms (30).

Chemotherapy-induced CRCI has been demonstrated in other malignancies (**Table 1**), including hematopoietic malignancies and testicular cancer (TC) (37, 38, 40, 41, 43, 68). Cancer survivors, including patients treated for non-Hodgkin’s and Hodgkin’s lymphoma, have cognitive impairment in domains including memory, learning and executive function for at least 6 months following chemotherapy compared to healthy controls (41, 42, 44). Twenty percent of TC patients report worsening cognitive impairment 2 years following multiagent bleomycin, etoposide and cisplatin (BEP) therapy using quality-of-life questionnaires (37, 69). Interestingly, TC patients treated with BEP did not have significant cognitive differences on neuropsychological testing from TC patients treated with radiation therapy and surgery (69, 70, 74, 75). BEP therapy has also been shown to cause significant brain networking changes and altered white matter microstructure in TC patients for durations up to 14 years after treatment compared to TC patients who did not receive chemotherapy (39, 73). Additional studies of TC survivors with long-term follow up for at least 2 years post treatment have found cognitive impairment following multiagent chemotherapy with or without ECRT compared to survivors treated with surgery alone (71, 72).

### 2.2 Radiation therapy

Extracranial radiation therapy (ECRT) is associated with cognitive impairment in cancer patients. There are substantial health implications of this phenomenon because over 50% of all newly diagnosed cancer patients will receive ECRT as part of their treatment protocol (7). Importantly, radiotherapy is prescribed in an estimated 56% of breast and prostate cancer patients (88, 89). Unsurprisingly, brain irradiation for primary or metastatic tumors causes brain damage leading to neurocognitive decline (5, 6). Somewhat less intuitively, cognitive impairment has been associated with ECRT directed to tumor-bearing anatomic sites outside the brain (5, 7, 45, 46). Little is known regarding the potential mechanism(s) linking ECRT and CRCI.

Similar to chemotherapy-associated CRCI, clinical evidence for ECRT-associated CRCI has arisen from BC survivors (**Table 1**). Studies have shown impairment in multiple cognitive domains in BC survivors after ECRT including complex cognition, attention, memory, and executive function (7, 20, 23). One study showed that 6 months following surgery and ECRT (no chemotherapy), BC survivors demonstrated higher rates of impairment in attention compared to age matched non-cancer control women (20). A separate study that assessed cognitive function via neuropsychological tests and self-reporting showed that 6 months after treatment, BC patients treated with ECRT only or chemotherapy and ECRT (chemoRT) had similar prevalence of cognitive impairment (47). This cognitive dysfunction is robust and lasts years following ECRT. BC patients treated with ECRT only or chemoRT sustained cognitive impairment at 1 year post ECRT (45). An additional longitudinal study found that BC patients treated with ECRT or chemoRT demonstrated worse cognitive performance compared to age matched non-cancer-bearing women in areas of executive function and processing speed up to 3 years post treatment (76). Similar to chemotherapy-induced CRCI, cognitive dysfunction has been documented in patients treated with ECRT for a variety of solid and hematologic tumor types (48). Moreover, this broad study supported that the impact of treatment extends well beyond resolution of acute radiation toxicities, as cancer patients treated with chemotherapy, ECRT or chemoRT reported problems with memory and concentration up to 6 months post treatment (48). ECRT-induced CRCI has also been documented in TC survivors (69, 71). Indeed, in one study with follow-up of TC survivors for over 20 years post treatment documented cognitive impairment with ECRT compared to age matched healthy controls (75).Similar to the proposed mechanisms underlying the contribution of chemotherapy to CRCI, it has been hypothesized that ECRT exerts its bystander cognitive impact through the circulatory trafficking of ECRT-induced pro-inflammatory cytokines to the brain that subsequently induce cognitive impairment (23, 46, 90, 91). Pro-inflammatory cytokines that are produced after ECRT include IL-1β, IL-1α, TNF-α, IL-6, IL-4, INF-ϒ, IL-8 (91, 92). IL-6 has been implicated as a potential cytokine target, as one study demonstrated memory deficits identified in BC patients up to 7 months post-ECRT were partially mediated by elevated plasma IL-6 levels (46).

### 2.3 Other cancer treatments

Chemotherapy and ECRT have garnered abundant attention as critical treatment modalities linked to CRCI. However, deleterious cognitive effects are reported with a diagnosis of cancer as well as with almost all cancer treatments, including surgery, endocrine therapy and immunotherapy (**Table 1**) (2, 93). Alarmingly, studies estimate that 40% of BC patients exhibit CRCI before the start of therapy; this may therefore be exacerbated with treatment (2). Early identification of patients at risk for CRCI, as well as knowledge of signals that contribute to CRCI, may aid in development of both preventive and mitigative strategies.

Endocrine therapy can cause cognitive impairment in cancer patients and appears to predominately affect the domains of verbal memory and processing speeds (2, 8, 94). Specific drugs associated with CRCI include aromatase inhibitors, selective estrogen receptor modulators/agonists and androgen deprivation therapy (8, 49, 77, 94, 95). The proposed mechanisms by which endocrine therapies cause CRCI are reduction in brain N-methy-D-aspartate receptor concentration, down regulation of brain derived neurotrophic factor expression, decreased antioxidant capacity of cells in the brain, and decreased amyloid β clearance (94). Use of tamoxifen, a commonly prescribed and important adjuvant therapy for women with hormone receptor positive BC, has been correlated with a reduction on measures of memory, verbal fluency, visuospatial functioning and processing speed (2, 77). In one study, women taking tamoxifen were more than twice as likely to report seeing a physician for memory issues as compared to BC patients not taking tamoxifen (96). Another study found that adjuvant hormonal therapy in BC patients has been associated with decreased verbal memory and processing speed (95). Tamoxifen may be given following chemotherapy or concurrent with chemotherapy, which may confound the effects of each individual drug. However, tamoxifen appears to play an important role in mediating CRCI as BC patients treated with tamoxifen and chemotherapy in one study had increased declines in visual memory and verbal working memory versus patients treated with chemotherapy alone (50). Prostate cancer patients treated with androgen deprivation therapies, including abiraterone acetate and enzalutamide, also report cognitive impairment (49, 94). One study found that patients with metastatic prostate cancer treated with enzalutamide had a significantly higher risk of clinically meaningful and worsening cognitive impairment than patients treated with abiraterone acetate plus prednisone up to 3 months after therapy (49).

Immunotherapies including cytokines, checkpoint inhibitors, chimeric antigen receptor (CAR) T cell therapies and hematopoietic stem cell transplantation have also been associated with CRCI (43, 51, 58, 59, 62, 94, 97). One study in patients with chronic myelogenous leukemia treated with interferon-alpha (INF-α) (with or without chemotherapy) showed declines in information processing and executive function when assessed by neuropsychological testing (51). This study also showed that 50% of the patients had significant declines in performance on one or more neuropsychological tests (51). Patients with melanoma, small cell lung cancer, non-small cell lung cancer and Merkel-cell carcinoma have exhibited neurotoxicity following immune checkpoint inhibitors five weeks after treatment (98). Approximately one-third of cancer patients had neurocognitive impairment after treatment with the popular immune checkpoint inhibitor pembrolizumab (52). Similar findings were found in a cross-sectional study, in which 41% of cancer survivors treated with the checkpoint inhibitor ipilimumab, had deficits in working memory and verbal memory (53). Cognitive impairment extends to CAR T-cell therapy as well, with 36% of 84 lymphoma patients treated with a CD19-targeted CAR T-cell developed severe cognitive impairment after treatment (54, 55). Up to 37% of patients with hematological neoplasms treated with CD-19 targeted CAR T-cell therapy reported cognitive impairment in one or more cognitive domains including memory, language, attention, and executive functioning, even up to 90 days after treatment (55, 56). Following allogeneic hematopoietic stem cell transplant for treatment of hematopoietic neoplasia, patients may demonstrate decline in verbal learning, reaction time, working memory, learning, executive function, attention, and psychomotor speed assessed by neuropsychological testing (58, 60, 61, 63, 78). In one study, up to 38% of patients with hematopoietic neoplasia had at least mild cognitive impairment 5 years following allogeneic bone marrow transplant (79). A separate study suggested that 41% of patients with hematopoietic neoplasia showed impairment in at least 1 cognitive domain one year post allogeneic bone marrow transplant (62).

Immunotherapies may contribute to CRCI via microglia-mediated neuronal death secondary to the production, circulation, and blood brain barrier penetration of proinflammatory cytokines (94). This supposition is supported by one study of non-CNS cancer patients demonstrating cognitive impairment that was correlated with pro-inflammatory plasma biomarkers including interferon-gamma (INF-ϒ), interleukin one beta (IL-1β), interleukin two (IL-2) and fibroblast growth factor two (FGF2) (57).

Clinical studies demonstrate serious cognitive impairment in cancer survivors that impacts quality-of-life measures for years following local and systemic treatments. Several treatments have been linked to changes in the brains of cancer patients, and to pro-inflammatory biomarkers in circulation. Future studies to identify reliable biomarkers may prove beneficial for detection, monitoring and response to treatment strategies aimed at preventing or mitigating CRCI.

## 3 CRCI animal models

Animal models of CRCI are critical to elucidating the mechanism(s) by which cancer treatments contribute to CRCI, and to the discovery of neuro-protective strategies that aim to improve quality of life (26, 99). The majority of preclinical studies use rodents including mice and rats to model CRCI in cancer patients (23, 99). In rodent studies of CRCI, experiments use a systemic approach to control for confounding variables in human cancer patients, including comorbidities, age, cancer type, disease progression, differences in baseline cognitive testing, and treatment regimens (26, 100). Through this approach, animal studies can account for age, sex, health status, and environment while also allowing for proper negative controls. Such rodent studies allowed for the assessment of the neurotoxic effects of a single cancer treatment agent, the characterization of potential mechanisms underlying cognitive deficits seen in cancer patients, and identified the cognitive domains affected by cancer treatments (23, 26). Preclinical studies have explored the cognitive and neurobiological effects of tumors with or without cancer treatments and investigated the effects of cancer treatment alone in healthy, tumor free rodents (23, 87, 101). Without question, selection of appropriate rodent models allow the acquisition of information that cannot be generated in human patients, thus addressing critical gaps in our knowledge of CRCI development.

### 3.1 Cancer drug treatment

Rodent studies have characterized the scope and mechanistic underpinnings of the cognitive impact of cancer treatments in both tumor-bearing and non-tumor bearing rodents. This has included studies of chemotherapy (single or multi-agent), ECRT, surgery, hormonal therapy and immunotherapy (5, 23, 26, 87, 93, 102, 103). Within the chemotherapy space, rodent studies have evaluated the effects of intravenous or intraperitoneal administration of alkylating agents (CYP, temozolomide, thiotepa), platinums (cisplatin, oxaliplatin, carboplatin), antimetabolites (MTX, 5-FU, cytosine arabinoside, cytarabine), anthracycline (DOX, epirubicin), antimicrotubule agents (vincristine, paclitaxel, DTX), cytosine arabinoside (cytarabine) and topoisomerase I inhibitor (topotecan) (5, 23, 26, 87, 99). Rodent studies investigating the mechanisms of ECRT induced CRCI, include ECRT models of non-CNS cancer treatment (5, 7, 12, 102). In this model, ECRT is represented by a single dose of ECRT to an extracranial region, such as the skin of the hindlimb, in tumor-free or tumor-bearing animals (5, 7, 12, 102).

### 3.2 Behavioral testing

Behavioral testing in rodents (**Table 2**) has bolstered the findings of long-lasting, potentially severe cognitive deficits following chemotherapy from observational studies of human cancer patients (15). Importantly, behavioral tests used in rodent CRCI studies evaluate similar cognitive domains as those reported to be affected in human CRCI studies, maximizing translational strength of these models. Testing in rodents evaluates memory, learning, executive function, attention and cognitive flexibility (5, 26, 99). In exploring the impact of chemotherapy on cognition in rodents, researchers have evaluated a wide variety of chemotherapy regimens in both tumor-free and tumor-bearing animals (23, 26). This work has revealed deficits in spatial working memory in rodents treated with cisplatin, vincristine, CYP, DOX, BCNU, topotecan MTX + 5-FU, CYP + DOX or CYP + DOX + 5-FU (99, 104–111, 152). Additionally, recognition/visual memory was reduced in rodents following single agent administration of cisplatin, DOX, MTX, 5-FU, CYP, DTX, thiotepa and oxaliplatin (OXP) (5, 100, 112–122). Multi-agent chemotherapy including CYP + DOX and OXP + 5-FU also resulted in impaired recognition/visual memory (123–127). Rodent learning was diminished after administration of several single and multiagent protocols, including cisplatin, BCNU, MTX, CYP, DOX, OXP, TMZ, MTX + 5-FU, CYP + DOX (128–138). Rodents have decreased executive function after treatment with the following chemotherapy agents MTX, 5-FU, cytarabine, and MTX + 5-FU (139–143). Some chemotherapy agents including carboplatin, DTX, CYP and topotecan also increase attention deficits (99, 144). Finally, cognitive flexibility appears to be impaired in rodents treated with 5-FU, MTX + cytarabine, paclitaxel and cisplatin (139, 145–148). Collectively, the broad and significant negative impact that systemic chemotherapy has on cognitive function in rodent models mirrors cognitive impairment described in pediatric and adult cancer patients.

**Table 2:** Summary of findings from pre-clinical studies evaluating cancer treatments effect on cognitive function.

Reference	Animal model		Treatment	Time post treatment	Behavioral effects
Seiger et al., 2015 (99)	C57BL/6J mice with no tumors		Single dose of 150mg/kg IP CYP	23 & 114 days 40 days	Hippocampal memory deficits Decreased inhibitory control
			Single dose of 33mg/kg IP DTX	23 & 114 days 40 days	Hippocampal memory deficits Decreased inhibitory control
			Single dose of 10mg/kg IV DOX	23 & 114 days 23 days 30 days 35 and 126 days	Hippocampal memory deficits Perirhinal cortex memory deficits Impairment in spatial working memory Increased associative memory
			Single dose of 5mg/kg IV DOX	114 days	Hippocampal memory deficits
			Single dose of 75mg/kg IP 5-FU	22 days 114 days 23 days 30 days	Anxiety like behavior Hippocampal memory deficits Perirhinal cortex memory deficits Impairment in spatial working memory
			Single dose of 250mg/kg IV MTX	114 days	Hippocampal memory deficits
			Single dose of 500mg/kg IV MTX	114 days	Hippocampal memory deficits
			Single dose of 25mg/kg IP Topotecan	23 & 114 days 23 days 30 days 40 days	Hippocampal memory deficits Perirhinal cortex memory deficits Impairment in spatial working memory Decreased inhibitory control
Shabani et al., 2012 (104)	Wistar rats without tumors		0.2mg/kg IP vincristine once a week for 5 weeks	1 day	Deficits in spatial learning and memory Suppressed motor activity and explorative behavior
Shabani et al., 2012 (105)	Wistar rats without tumors		5mg/kg IP cisplatin once a week for 5 weeks	1 day	Decreased locomotor activity Impairment in spatial working memory
Winocur et al., 2015 (106)	Long-Evans rats without tumors		37.5mg/kg IP MTX and 50mg/kg IP once a week for 3 weeks	5 weeks	Impairment in hippocampal dependent spatial memory
Winocur et al., 2014 (107)	Long-Evans rats without tumors		37.5mg/kg IP MTX and 50mg/kg IP 5-FU once a week for 3 weeks	1 weeks	Impairment in hippocampal dependent spatial working memory
Winocur et al., 2006 (108)	Balb/c mice without tumors		37.5mg/kg IP MTX and 75mg/kg IP 5-FU once a week for 3 weeks	1 weeks	Impairment in spatial learning and working memory
Inverardi et al., 2013 (109)	Sprague-Dawley rats without tumors		Single dose of 20mg/kg IP BCNU to pregnant rats	Pups were tested at 3-5 months of age	Impairment in short-term working memory
Janelsins et al., 2016 (110)	C57BL/6 mice without tumors		200mg/kg IV CYP once a week for 4 weeks	3 months	Deficits in spatial working memory
Renderio et al., 2016 (111)	C57BL/6 mice without tumors		4mg/kg IV DOX, 80mg/kg IP CYP and 5-FU 40mg/kg IP once a week for 2 weeks	3 months	Deficits in spatial learning and working memory
Christie et al., 2012 (100)	Athymic nude rats without tumors		50mg/kg IP CYP once a week for 4 weeks	1 week 2 weeks	Impairment in hippocampal-based spatial recognition memory Impairment in hippocampal dependent contextual memory
			2mg/kg IP DOX once a week for 4 weeks	1 week 2 weeks	Impairment in hippocampal-based spatial recognition memory Impairment in hippocampal dependent contextual memory
Lyons et al., 2011 (112)	Lister hooded rats without tumors		75mg/kg IV MTX once a week for 2 weeks	1 week	Impairment in spatial recognition memory
Vijayanathan et al., 2011 (113)	Long Evans rats without tumors		4 doses of 0.5mg/kg IT MTX over 10 days	3 months	Impairment in recognition and spatial memory
			Single dose of 0.5mg/kg IT MTX	1 week	Impairment in working memory
Elbeltagy et al., 2011 (114)	Lister hooded rats without tumors		Six doses of 20mg/kg IV 5-FU once every other day for 2 weeks	1 day	Impairment in spatial recognition memory
Lyons et al., 2012 (115)	Lister hooded rats without tumors		25mg/kg IP 5-FU given every 3 days for a total of 5 doses	27 days	Impairment in spatial recognition memory
Mustafa at al. 2008 (116)	Lister hooded rats without tumors		5 doses of 20mg/kg IV 5-FU given over 12 days	1 day	Impairment in spatial recognition memory
Acharya et al., 2015 (117)	Athymic nude rate without tumors		100mg/kg IP CYP once a week for 4 weeks	5 weeks	Impairment in hippocampal learning and recognition memory
Callaghan et al., 2015 (118)	Han Wistar rats without tumors		1mg/kg IV DTX once a week for 4 weeks	8 weeks	Impairment in hippocampal-dependent spatial memory
Fardell et al., 2015 (119)	Hooded Wistar rats without tumors		6mg/kg IP OXP once a week for 3 weeks	11 months	Impairment in location recognition Impairment in object recognition
			2mg/kg IP OXP once a week for 3 weeks	1 month	Impairment in object recognition
			0.6mg/kg IP OXP once a week for 3 weeks	1 month	Impairment in object recognition
Fardell et al., 2013 (120)	Hooded Wistar rats without tumors		6mg/kg IP DTX once a week for 3 weeks	9 days 112 days	Impairment in spatial recognition memory Impairment in spatial learning and reference memory
			10mg/kg IP DTX once a week for 3 weeks	9 days 112 days	Impairment in spatial recognition memory Impairment in spatial learning and reference memory
			Single dose of 10mg/kg IP DTX	21 days	Impairment in spatial recognition memory
Lomeli et al., 2017 (121)	Sprague Dawley rats without tumors		5mg/kg IP cisplatin once a week for 4 weeks	5-6 weeks	Impairment in context memory Impairment in spatial recognition memory
Mondie et al., 2010 (122)	C57BL/6J without tumors		10mg/kg IP thiotepa for 3 consecutive days	8-12 weeks 20 weeks	Deficits in object recognition memory Deficits in spatial memory
Kitamura et al., 2015 (123)	Wistar rats without tumors		2mg/kg IP DOX and 50mg/kg IP CYP once a week for 4 weeks	1 week	Deficits in spatial cognition Induction of anxiety like behavior
			2mg/kg IP DOX once a week for 4 weeks	1 week	Deficit in spatial cognition Induction of anxiety like behavior
			50mg/kg IP CYP once a week for 4 weeks	1 week	Deficits in spatial cognition Induction of anxiety like behavior
Kitamura et al., 2017 (124)	Wistar rats without tumors		2mg/kg IP DOX and 50mg/kg IP CYP once a week for 4 weeks	1 week	Induction of anxiety like behavior Deficits in spatial cognition Impairment of working memory
Salas-Ramirez et al., 2015 (125)	Sprague-Dawley rats without tumors		40mg/kg IV CYP and 4mg/kg IV DOX once a week for 3 weeks	1 week	Impairment of working memory Impairment of spatial memory
Iarkov et al., 2016 (126)	Wistar rats without tumors		40mg/kg IP CYP, 37.5mg/kg IP MXT and 75mg/kg IP 5-FU once a week for 2 weeks	3 weeks	Impairment in spatial working memory
Fardell et al., 2012 (127)	Hooded Wistar rats without tumors		Single dose of 12mg/kg IP OXP	14 days	Impaired in recognition memory
			Single dose of 75mg/kg IP 5-FU	14 days	Impaired in recognition memory
			Single dose of 12mg/kg IP OXP and 75mg/kg IP 5-FU	14 days 28 days	Impaired in recognition memory Impairment in contextual memory recall
Winocur et al., 2012 (128)	Balb/c mice without tumors		37.5mg/kg IP MTX and 50mg/kg IP 5-FU once a week for 3 weeks	1 weeks 3 months	Impairment in hippocampal and frontal lobe dependent memory Impairment in hippocampal dependent spatial working memory
Nokia et al., 2012 (129)	Sprague-Dawley rats without tumors		25mg/kg IP TMZ once per day for 3 consecutive days per week for 5 weeks	1 day	Decrease in attention Impairment in hippocampal active learning
Madhyastha et al., 2002 (130)	Wister rat without tumors		3 doses of 1.5mg/kg ICV MTX	1 week	Impairment of learning and memory
			3 doses of 2mg/kg ICV MTX	1 week	Reduction in locomotor and exploratory activity Impairment of learning and memory
Song et al., 2010 (131)	CBA mice without tumors		5mg/kg IP cisplatin given every other day for a total of 3 doses	31 and 45 days 7 days	Impairment in memory retention Impairment in learning
Seigers et al., 2009 (132)	Wister rats without tumors		Single dose of 250mg/kg IV MTX	1 week and 1 month	Impairment in learning and memory
Yang et al., 2012 (133)	C3H/HeN mice with tumor (mammary carcinoma) on right flank		One week after tumor inoculation given a single dose of 40mg/kg IP MTX	1 day	Increased depressive like behavior Impaired hippocampal-related learning
Bianchi et al., 2017 (134)	Rats without tumors		2.4mg/kg IP OXP once a day for 5 days every week for 2 weeks	3 weeks	Impairment in social learning and passive avoidance
Hou J-g et al., 2013 (135)	ICR mice without tumors		80mg/kg IP CYP once a week for 4 weeks	1 day	Impairment in spatial memory and learning
Lim et al., 2016 (136)	Sprague-Dawley rats without tumors		100mg/kg IP CYP	1 day	Impairment in spatial learning ability and reference memory Impairment in working memory
			4mg/kg IP DOX once a week for 3 weeks	1 day	Impairment in spatial learning ability and reference memory Impairment in working memory
Konat et al., 2008 (137)	Sprague-Dawley rats without tumors		2.5mg/kg IP DOX and 25mg/kg IP CYP once a week for 4 weeks	2 days	Impairment in short-term memory and learning
Helal et al., 2009 (138)	Wistar albino rats without tumors		Single dose of 20mg/kg IV BCNU	3 weeks	Impairment in learning and short-term memory
Bisen-Hersh et al., 2013 (139)	Swiss-Webster mice without tumors		1mg/kg IP MTX once a day for 3 days	18 days 19 days	Impairment in response discrimination Impairment in retention and acquisition
			2mg/kg IP MTX once a day for 3 days	18 days 19 days	Impairment in response discrimination Impairment in recognition memory Impairment in retention and acquisition
			10mg/kg IP cytarabine once a day for 3 days	18 days 19 days	Impairment in response discrimination Impairment in retention and acquisition
			20mg/kg IP cytarabine once a day for 3 days	18 days 19 days	Impairment in response discrimination Impairment in recognition memory Impairment in retention and acquisition
			1mg/kg IP MTX and 10mg/kg IP cytarabine once a day for 3 days	18 days 19 days	Impairment in response discrimination Impairment in retention and acquisition
			2mg/kg IP MTX and 20mg/kg IP cytarabine once a day for 3 days	18 days 19 days	Impairment in response discrimination Impairment in recognition memory Impairment in retention and acquisition
Foley et al., 2008 (140)	Swiss-Webster mice without tumors		Single dose of 75mg/kg IP 5-FU	1 day	Impairment in learning and memory
			Single dose of 3.2mg/kg IP MTX and 75mg/kg IP 5-FU	1 day	Impairment in learning and memory
Walker et al., 2011 (141)	Swiss-Webster mice without tumors		Single dose of 32mg/kg IP tamoxifen	1 day	Impairment in learning and memory
			Single dose of 3.2mg/kg IP MTX and 75mg/kg IP 5-FU	1 days	Impairment in learning and memory
Krynetskiy et al., 2013 (142)	Swiss-Webster mice without tumors		3 doses of 75mg/kg IP 5-FU over one day	1 day	Impairment in learning acquisition and memory retention
Fardell et al., 2010 (143)	Hooded Wistar rats without tumors		Single dose of 250mg/kg IP MTX	11 day, 95 days 95 days 174 days	Impairment in recognition memory Impairment in long-term memory Impairment in instrumental long term learning
Kaplan et al., 2016 (144)	Wistar rats without tumors		20mg/kg IV carboplatin once a week for 4 weeks	24 hours	Impairment in spatial learning, visual attention and memory
Dubois et al., 2014 (145)	C57BL/6J mice without tumors		Single dose 37.5mg/kg IP 5-FU every 7 days for 3 weeks	5 weeks	Deficits in executive function
Smith et al., 2017 (146)	Long Evans rats without tumors		2mg/kg IP paclitaxel every day for 4 days	8 weeks	Impairment in learning
Mu et al., 2015 (147)	Sprague-Dawley rats without tumors		5mg/kg IP cisplatin once a week for 6 weeks	After 3^rd^ dose of cisplatin 22 days after final dose	Anxiety like behavior Impairment in spatial reference memory Impairment in behavioral flexibility
			5mg/kg IP cisplatin once a week for 8 weeks	After 3^rd^ dose of cisplatin 1 day after last injection	Anxiety like behavior Impairment in spatial reference memory
Chui et al., 2017 (148)	C57BL/6J mice without tumors		2.3mg/kg IP cisplatin once a day for 5 days	7 days	Impairment in spatial, recognition and working memory
Demos-Davies et al., 2022 (5)	SKH1 mice with no tumors		Single dose of 20Gy ECRT	14 days	Hippocampal dependent memory deficits
			Single dose of 5 mg/kg IP DOX	14 days	Hippocampal and cortical region recognition memory deficits
			Single dose of 20Gy ECRT and 5mg/kg IP DOX	14 days	Hippocampal dependent memory deficits
McGinnis et al., 2017 (102)	BALB/C mice without tumors		250ug IP Anti-CLTA4 antibody followed by 20Gy ECRT one week later	Day 4 after ECRT	Impairment in recognition memory
	BALB/C mice with tumor (colorectal carcinoma) on right flank		Single dose of 250ug IP Anti-CLTA4 antibody	Day 4 after ECRT	Impairment in recognition memory
	BALB/C mice with tumor (colorectal carcinoma) on right flank		Single dose of 250ug IP Anti-CLTA4 antibody followed by 20Gy ECRT one week later	Day 4 after ECRT	Impairment in recognition memory
	C57BL/6 mice without tumors		Single dose of 250ug IP Anti-CLTA4 antibody followed by 20Gy ECRT one week later	Day 4 after ECRT	Impairment in recognition memory
	C57BL/6 mice without tumor (lung adenocarcinoma) on right flank		Single dose of 250ug IP Anti-CLTA4 antibody	Day 4 after ECRT	Impairment in recognition memory
	C57BL/6 mice without tumor (lung adenocarcinoma) on right flank		Single dose of 50ug IP Anti-CLTA4 antibody followed by 20Gy ECRT one week later	Day 4 after ECRT	Impairment in recognition memory Impairment of spatial recognition memory
Chen et al., 2002 (149)	Swiss mice		Single dose of 1mg/kg IP tamoxifen	30 minutes	Impairment in memory consolidation processes
			Single dose of 10mg/kg IP tamoxifen	30 minutes	Impairment in memory consolidation and retrieval processes
			Single dose of 3mg/kg IP toremifene	30 minutes	Impairment in memory acquisition processes
			Single dose of 10mg/kg IP toremifene	30 minutes	Impairment in memory acquisition and retrieval processes
			Single dose of 30mg/kg IP toremifene	30 minutes	Impairment in memory acquisition and consolidation processes
Chen et al., 2002 (150)	Swiss mice		Single dose of 1mg/kg IP tamoxifen	30 minutes	Impairment of spatial memory
			Single dose of 3mg/kg IP tamoxifen	30 minutes	Impairment of spatial memory
			Single dose of 10mg/kg IP tamoxifen	30 minutes	Impairment of spatial memory
			Single dose of 3mg/kg IP toremifene	30 minutes	Impairment of spatial memory
			Single dose of 10mg/kg IP toremifene	30 minutes	Impairment of spatial memory
			Single dose of 30mg/kg IP toremifene	30 minutes	Impairment of spatial memory
Esmaeili et al., 2009 (151)	NMRI mice		Single dose of 10mg/kg SC tamoxifen	30 minutes	Disrupt retrieval of morphine-associated contextual memory
Chen et al., 2023 (93)	SD rats		3% sevoflurane for 2 hours	7 days	Anxiety like behavior Impairment in spatial learning and recognition memory

IP, intraperitoneal; IV, intravenous; SC, subcutaneous; IT intrathecal; ICV, intracerebroventricular; ECRT, extracranial radiation therapy; DOX, doxorubicin; CYP, cyclophosphamide; MTX, methotrexate; 5-FU, 5-floururacil; DTX, docetaxel; CMF, cyclophosphamide, methotrexate, 5-floururacil; BEP, bleomycin, etoposide, cisplatin; OXP, oxaliplatin; BCNU, carmustine.

The impact of ECRT on cognition has been less thoroughly explored in rodent models but is of recent interest. Most notably, one study demonstrated that hippocampal dependent memory was impaired in mice treated with ECRT as compared to untreated control mice (5). Importantly, the memory deficits in these mice were comparable to mice treated with DOX and DOX + ECRT (5). The results of this study complement those reported in a carcinoma-bearing mouse model in which combination immunotherapy and ECRT lead to impairment in visual/recognition memory as compared to non-treated, tumor free control mice (102).

Hormonal therapy in intact rodents has also been shown to negatively impact memory, learning and executive functioning (103, 141). One study found that tumor-free mice treated with tamoxifen, MTX and/or 5-FU had compromised learning and executive function as compared to untreated control mice (141). This work parallels other studies that have shown that tamoxifen alone can cause impairment in memory, learning and executive function (149–151). Finally, surgical anesthesia has been shown to induce deficits in working memory and recognition/visual memory (93). A study reported that rats anesthetized with sevoflurane inhalant demonstrate diminished working and recognition memory as compared to untreated control rats (93).

### 3.3 Neuropathological changes

While the descriptive confirmation of the impact of cancer treatment on the development of CRCI has been well documented in rodent models, studies have also provided invaluable insights into the mechanistic links between treatment and CRCI. Such work has shown that cancer treatments result in reduced neurogenesis, neuroinflammation, reduced brain glucose metabolism, alterations in brain neurotransmitter levels, decreased brain myelination, detrimental changes in neuron axons and dendrites, increased cellular damage in the hippocampus, reduction of white matter integrity, DNA damage, oxidative stress and mitochondrial dysfunction in the hippocampus (26, 121, 134, 148).

Multiple studies have positively correlated the behavioral deficits observed in treated mice to pathological changes within the brain (106, 142). One study demonstrated a positive correlation between DNA damage in the brain cells of mice treated with 5-FU and the learning deficit seen in these mice (142). Another study found in rats treated with 5-FU and methotrexate a significant correlation between decreased neurogenesis in the hippocampus of these rats and impairment of hippocampal memory (106). Similar associations have been found in BC patients treated with chemotherapy (4).

The impact of single-agent and multi-agent chemotherapy on neurogenesis, particularly within the hippocampus, is well-documented (26, 87, 153). Specifically, 14 independent rodent studies have shown that treatment with BCNU, MTX, 5-FU, CYP, DOX, TMZ, cisplatin, thiotepa, DOX + CYP, MTX+5-FU and CYP + DOX + 5-FU results in decreased neurogenesis in the hippocampal dentate gyrus as compared to non-chemotherapy treated rodents (100, 106, 111, 112, 122, 124, 129, 132, 135, 145, 148, 154–156). One study in rats expanded upon these findings and demonstrated that treatment with MTX+5-FU resulted in detectable cognitive dysfunction that correlated with reduced hippocampal neurogenesis (106). It is not yet clear how systemic chemotherapy impacts neurogenesis. Decreased levels of hippocampal brain-derived neurotropic factor and doublecortin may disrupt neurogenesis, based on one study in rats following 5-FU administration (116). Reduced neurogenesis following chemotherapy is one of several potential mechanisms by which chemotherapy alters cognition. This occurrence has profound implications in human patients because reduced neurogenesis may also contribute to depression and accelerate cognitive diseases like Alzheimer’s (157).

There is also robust evidence that cancer treatments induce neuroinflammation, which can lead to cognitive dysfunction itself while also contributing to reduced neurogenesis. Numerous studies have documented neuroinflammation or upregulation of brain proinflammatory cytokines, or both in rodents treated with chemotherapy, ECRT, immunotherapy or combination therapy (5, 100, 158). Specific chemotherapy agents that cause neuroinflammation in rodents include CYP, DOX and MTX (100, 133, 159). Two recent murine studies have also shown that hindlimb irradiation with appropriate blocking of CNS tissue, results in widespread brain astrocytosis and microgliosis (components of neuroinflammation) up to 30 days after ECRT treatment compared to control mice (5, 7). A similar finding was described in both tumor-free and tumor-bearing mice treated with immunotherapy or hindlimb irradiation or both had increased activated microglia in the hippocampus and cortex as compared to untreated control mice (102). These studies are complemented by work documenting increased levels of pro-inflammatory reactive oxygen species (ROS) in rodent models of CRCI. Cancer treatments in rodents increase ROS characterized by increased oxidative stress and DNA damage (87). The chemotherapy agents cisplatin, carboplatin, CYP, BCNU, cytarabine, DOX, and MTX have specifically been shown to increase brain oxidative stress (138, 160–168). Of import to cancer patients undergoing surgical procedures, the anesthetic agent sevoflurane has also been shown to cause increased hippocampal oxidative stress in rats (93).

Metabolic derangement has been highlighted in other studies as an important potential contributing factor to CRCI. Decreased brain glucose metabolism has been observed during FDG (fluorodeoxyglucose)-PET scan in rodents treated with either chemotherapy or ECRT (7, 136, 159, 169). Rats treated with CYP or DOX in one study showed decreased glucose metabolism in the medial cortex with both treatments and in the hippocampus in the DOX-treated rats (136). Similar findings were seen in a study in which DOX-treated rats had decreased prefrontal cortical glucose metabolism 30 days post treatment compared to untreated controls (169). MTX treatment to rats also caused reduced hippocampal glucose metabolism with decreased blood vessel density compared to untreated rats (159). Mice treated with hindlimb irradiation alone also developed reduced brain glucose metabolism after treatment compared to their pre-ECRT brain imaging (7).

Alterations in neurotransmitter levels and neurotransmission has been shown in the brains of rodents treated with MTX, 5-FU, carboplatin and DOX (6, 130, 144, 170, 171). Decreased levels of hippocampal norepinephrine, dopamine and serotonin were measured following MTX administration (130). Additionally, impaired dopamine release and update occurred following carboplatin and 5-FU administration in rats (144, 170). In mice, treatment with DOX results in impaired glutamatergic signaling (171). Finally, changes in the structures of rodent neuronal dendrites and axons have been described after chemotherapy (26, 121). Specifically, CYP, cisplatin and cytarabine have shown to reduce dendritic length, volume, spine density and number of branch points (26, 100, 117, 172). Cisplatin given to rats has also resulted in reduced hippocampal pyramidal neuron dendritic branching and spine density up to 28 days after administration (121).

Collectively, animal studies have demonstrated impairment in cognitive domains similar to cancer survivors after systemic and local antineoplastic treatment. Cognitive impairment has been correlated to neuropathological findings in the brain of rodents, which has led to the identification of potential mechanisms of cancer therapy-induced CRCI. Mechanisms implicated in the cause of CRCI include decreased neurotransmission, disruption of the blood brain barrier, impaired neurogenesis, increased neuronal oxidative stress, neuroinflammation, and decreased cerebral blood flow (87). Decreased neurotransmission is thought to be due to pro-inflammatory cytokines such as IL-1β that can lead to the catabolism of neurotransmitters and dysregulation of neurotransmission that can cause cognitive impairment (13, 87). Disruption of the blood brain barrier via peripheral cytokine dysregulation can lead to neuroinflammation causing cognitive impairment (13). Rodent animal models have highlighted that cancer treatments decreases neurogenesis through the production of pro-inflammatory cytokines such as TNF-α and subsequent activation of glial cells (22, 87, 94). Most cancer treatments induce the production of reactive oxygen species and oxidative stress, which in turn leads to neuronal DNA damage (22, 87, 91, 94). Pro-inflammatory cytokines produced in the tumor microenvironment can enter circulation and cross the blood brain barrier, leading to the activation of glial cells (13). Activated glial cells contribute to neuron damage and cognitive impairment (6, 173). Finally, several preclinical models have shown that cancer treatment can reduce cerebral blood flow and glucose metabolism, further contributing to cognitive impairment (7, 87, 94, 174). Importantly, most of the proposed mechanisms include a significant initial contribution from pro-inflammatory mediators. Further work in animal models is needed to target these pro-inflammatory mediators in attempts to elucidate new approaches that may mitigate CRCI.

## 4 Conclusion and future directions

Quality of life for cancer survivors deserves the spotlight of attention because of its widespread impact on psychosocial and economic well-being. Published literature clearly and repeatedly documents that cancer therapy results in cognitive impairment that persists for years after treatment. However, there are limitations in interpreting published studies in cancer patients due to heterogeneity in patient populations, differences in treatment regimens, a lack of standardized neuropsychologic testing approach, wide variability in the control patient population and lack of assessing the cognitive impact of a cancer diagnosis over time (20, 25, 33, 35, 45–48, 76). These limitations with interpretation of existing data serve as a platform upon which to build prospective preclinical and clinical studies. Future clinical studies should include standardized assessment of cognitive function prior to treatment, during therapy, and with serial monitoring protocols, ideally for the life of the patient. The addition of biomarkers, such as known pro-inflammatory serum cytokines, and functional imaging studies may be a helpful addition to established cognitive function protocols. These studies would provide critical data to aid in the design of optimal cognitive monitoring. The Common Terminology Criteria for Adverse Events (CTCAE) v5.0 guidelines currently includes monitoring for cognitive dysfunction. However, as studies isolate specific proinflammatory mediators or distinguish types of dysfunctions, future versions of CTCAE may opt to include measures for improved monitoring.

Because studies in cancer survivors are often limited by multiple confounding variables, animal models are essential to investigate the neurobiological mechanism by which cancer treatment disrupts the brain. Collectively, CRCI animal models support multiple factors including reduced neurogenesis, neuroinflammation, metabolic derangements, DNA damage, oxidative stress and altered neurotransmission create a cycle of signals to promote cognitive dysfunction (87, 93, 138, 160–168). These studies have provided insight into potential molecular processes by identifying protein and gene differentially expressed in the brain of rodents treated with cancer therapies used in human oncology. Future studies are needed to evaluate equivalent dosing strategies to human cancer therapy including using multi-agent treatment since most cancer patients are exposed to more than one type of anti-cancer therapy (5, 7). Identification of critical signaling disruptions that occur with therapy is vital to the development of effective preventive and mitigative strategies that allow cancer survivors to maintain high cognitive function (5).

## Author contributions

KD-D: Writing – original draft, Writing – review & editing. JL: Writing – original draft, Writing – review & editing. DS: Writing – original draft, Writing – review & editing.
